# To the Patrons of the Journal—End of Volume II; Beginning of Volume III

**Published:** 1887-06

**Authors:** 


### EDITORIAL DEPARTMENT.

F. E. DANIEL, M. D., EDITOR AND PUBLISHER.

COLLABORATORS:

E. J. Doering, M. D., Chicago.

Geo. Cupples, M. D., San Antonio.

Odo Betz, M. D., Germany.

E. J. Beall, M. D., Fort Worth, Texas.

T. C. Osborn, M. D., Cleburne, Texas.

Wm. Penny, M. D., New York.

H. O. Marcy, M. D., Boston.

C. K. Gregg, M. D., Mexico.

R. M. Swearingen, M. D., Austin.

C. H. Wilkinson, M. D., Galveston.

J. W. McLaughlin, M. D., Austin.

R. H. L. Bibb, M. D., Mexico.

This number completes Volume II. A neat title page and complete index accompany it.

On the eve of the third year of a successful career, the JOURNAL sends greeting to its hosts of friends and patrons; and gratefully acknowledging that generous support, both moral and pecuniary, so liberally bestowed by an appreciative profession, and to which its success is due, earnestly solicits a continuation of their favors.

It is gratifying to announce that what was, two years ago, an *experiment*, is now a *valuable property.*

Volume 3 begins July 1st, prox., under the most favorable auspices, and with greatly increased patronage and enlarged facilities. It is proposed to increase the monthly edition to 2000 copies—*just double* that of the past—at an early day, perhaps with the very next number; while the number of pages devoted to original reading matter will be increased one third; and the book enlarged from 60 to 86 pages. Original translations from the French and German Journals, and an occasional portrait of some prominent Texas physician will be the new feature added, all of which will make it even more attractive, and the best Journal for the price, published in the great South-west. Now is the time to subscribe; don't delay: begin with the new volume; and, dear reader, let us suggest, that it is *the best time in the world to settle up for last years subscription.* The subscription of many expired June, 1886, just one year ago, and next number, as said, begins another round, when two years subscription will be due. It is easier to pay two dollars than to pay four dollars—a hint to the wise. We will appreciate the courtesy of a prompt remittance from any and all whom the "cap fits."

The JOURNAL wishes you all a happy and prosperous year.

